# Effects of Subacute Hypothyroidism on Metabolism and Growth-Related Molecules

**DOI:** 10.3390/molecules190811178

**Published:** 2014-07-30

**Authors:** Yen-Jui Chang, Chii-Min Hwu, Chii-Chang Yeh, Paulus S. Wang, Shyi-Wu Wang

**Affiliations:** 1 Department of Physiology, School of Medicine, National Yang-Ming University, Taipei 11221, Taiwan; 2 Department of Surgery, Yangming Branch, Taipei City Hospital, Taipei 11146, Taiwan; 3 Departments of Internal Medicine and Medical Research and Education, Taipei Veterans General Hospital, Taipei 11217, Taiwan; 4 Department of Internal Medicine, Yangming Branch, Taipei City Hospital, Taipei 11146, Taiwan; 5 Department of Medical Research and Education, Taipei Veterans General Hospital, Taipei 11217, Taiwan; 6 Medical Center of Aging Research, China Medical University Hospital, Taichung 40402, Taiwan; 7 Department of Biotechnology, College of Health Science, Asia University, Taichung 41354, Taiwan; 8 Department of Physiology and Pharmacology, Chang Gung University, Taoyuan 33333, Taiwan

**Keywords:** hypothyroidism, thyroxine, growth-related molecules, ghrelin, insulin-like growth factor-1, growth hormone

## Abstract

Thyroid hormones are crucial hormones that primarily regulate the metabolism of entire body cells. In this study, Sprague-Dawley rats were grouped into sham thyroidectomy (Sham Tx), thyroidectomy (Tx), Tx with thyroxine replacement (Tx + T4), and PTU injection (PTU) groups. Metabolic parameters were measured by means of metabolic cages for 14 days. After 14 days, the rats were sacrificed while the levels of plasma or serum TSH and growth-related molecules, such as active and total ghrelin, GH, and IGF-1, were assayed. The results revealed that hypothyroid rats tended to eat less food and experienced substantial body weight gain, whereas the rats with T4 replacement tended to eat more food while continuing to lose weight. In hypothyroid rats, the growth-related molecules, such as active ghrelin and total ghrelin secretion, were enhanced, and the ghrelin receptors were also up-regulated. However, circulating GH levels were not elevated and IGF-1 secretion was inhibited in hypothyroid rats. In the Tx + T4 group, the changes of active ghrelin, total ghrelin, GHS-R expression, and IGF-1 were reversed, whereas the GH secretion was higher than that of the Sham Tx group and hypothyroid groups. This study resulted in the novel finding that the ghrelin/GHS-R axis and GH/IGF-1 axis are interrupted in hypothyroid rats.

## 1. Introduction

Thyroid hormones are secreted by follicular cells in the thyroid gland. A thyroid-stimulating hormone (TSH) stimulates the secretion of thyroid hormones, including triiodothyronine (T_3_) and thyroxine (T_4_). In plasma, the ratio of T_4_ and T_3_ is approximately 20:1. The half-life of T_4_ is longer than that of T_3_. Thyroxine is converted to T_3_ in peripheral tissue by deiodinases (5'-iodinase). However, the potency of T_3_ is three to four times that of T_4_. T_3_ and T_4_ can be decarboxylated or deiodinated to produce iodothyronamine (T_1_a) and thyronamine (T_0_a) [1,2]. Thyroid hormones act on almost all cells in the body with various functions, including increasing the basal metabolic rate, affecting protein synthesis, and regulating the metabolism of protein, lipids, and carbohydrates. Through synergy with the growth hormone, thyroid hormones modulate the growth of long bones and the differentiation of neurons. They also enhance tissue sensitivity to catecholamines, such as adrenalin and noradrenalin [3].

Thyroid dysfunctions may also have metabolic consequences. They can be divided into hyperthyroidism and hypothyroidism. In contrast to hypothyroidism, the typical symptoms of hyperthyroidism are increased basal metabolic rate, body weight loss, and tachycardia. Various animal models for thyroid dysfunctions have been used in rats. The thyroid glands are resected through thyroidectomy or destroyed by propylthiouracil (PTU) and methimazole. The circulating thyroid hormones are metabolized and cleaned after 2 weeks. Rats without thyroid hormone replacement exhibit hypothyroidism, and those administered 300 μg/kg or more of T_4_ exhibit hyperthyroidism [4]. Thyroid hormones are administrated subcutaneously, intraperitoneally, or orally [5,6,7,8].

The surgical removal of the thyroid gland involves the removal of the calcitonin-secreting parafollicular cells and the parathyroid gland. Therefore, it alters the homeostasis of circulating calcium. Conversely, chemical destruction of the thyroid gland by PTU alters hematopoiesis, the secretion of gastric inhibitory peptide, and steroidogenesis [9,10,11,12]. We used both surgical and chemical methods to destroy the thyroid gland tissue in order to examine the effects of hypothyroidism.

Several circulating molecules are involved in physical growth. Smith *et al.* found that small synthetic molecules can stimulate the secretion of growth hormones. These molecules bind to a G-protein-coupled receptor called the growth-hormone secretagogue receptor (GHS-R) to achieve this effect [13]. Kojim *et al.* isolated a molecule from the stomachs of rats that specifically binds to GHS-R and has the most potent stimulating effect on growth hormone secretion among those molecules. This endogenous ligand to GHS-R is called ghrelin [14]. Ghrelin is composed of 28 amino acids and has two major forms: active *n*-octanoyl-modified ghrelin and inactive des-acyl ghrelin. The active ghrelin is *n*-octanoyl-modified in serine 3. Only active ghrelin has bioactivities [15] that trigger the secretion of growth hormones and enhance appetite [16,17,18]. The growth hormone stimulates the production of insulin-like growth factor-1 (IGF-1) in hepatocytes. IGF-1 has growth-promoting effects on almost all cells in the body, particularly skeletal muscle, bone, cartilage, liver, kidney, nerve, skin, hematopoietic tissue, and lung cells [19].

Plasma ghrelin levels may be altered through various factors, such as diet [20,21] or environmental hormones [22]. Previous studies have indicated that thyroid dysfunctions alter the secretion and clearance of other hormones, such as the luteinizing hormone (LH) [23], gonadotropin-releasing hormone (GnRH), prolactin [24], insulin, and gastric inhibitory polypeptide [7]. Several studies have indicated that ghrelin levels in the blood decrease in hyperthyroid patients. The ghrelin levels return to the reference range after anti-hyperthyroid medicine treatments [25,26]. By contrast, ghrelin levels increase in hypothyroid patients [27]. However, previous studies have not examined the effects of thyroid dysfunction on GHS-R in the hypothalamus and the anterior pituitary gland. The alteration of ghrelin may also affect the secretion and interactions of downstream substances, growth hormones (GH), and IGF-1.

In this study, we measured the metabolic parameters of rats, such as body weight, food intake, feces output, water consumption, and urine output, as well as active ghrelin and total ghrelin secretion in circulation and GHS-R in the anterior pituitary gland and hypothalamus. Furthermore, the circulating downstream hormones GH and IGF-1 were also measured to investigate the changes under hypothyroid conditions.

## 2. Results and Discussion

### 2.1. Changes in Body Weight and Metabolism

**Figure 1:**
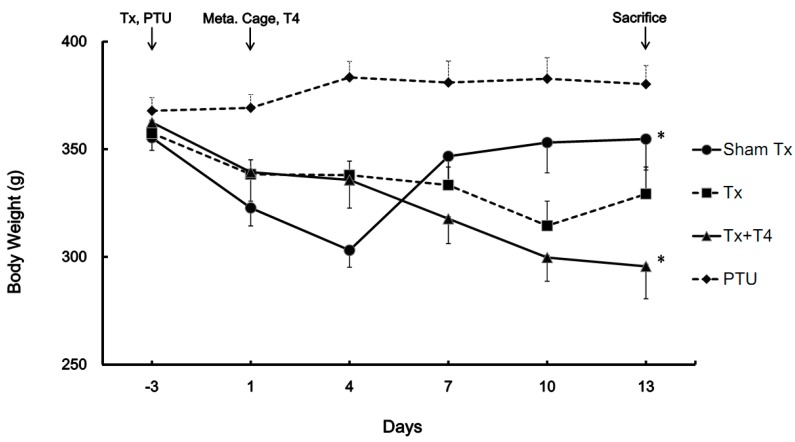
The changes of body weight in rats (N = 8). After surgeries, all rats were allowed to rest for 3 days. The rats in the PTU group were injected with 20 mg/kg of PTU since Day minus 3. After a 3 days resting period, all rats were housed in metabolic cages for investigation. The Sham Tx and Tx groups were injected with 1 mL/kg alkaline saline for 14 days. Rats in the Tx + T4 group were injected with 20 μg T_4_/kg for 14 days. On Day 1, lower body weight in the Sham Tx, Tx, and Tx + T4 groups than that in the PTU group was observed because of the operation conducted 3 days prior. The BW of the Sham Tx group increased considerably compared with that on Day 1. The BW of the Tx group decreased during the first 10 days and subsequently increased. The BW of the Tx + T4 group decreased during the experimental period. The PTU group maintained their body weight without any remarkable increase. * *p* < 0.05 compared with the value of Day 1 of each group.

The body weights of rats during the 2 weeks housing period in metabolic cages in each group are shown in Figure 1. In the Sham Tx group, the body weights increased gradually from 323 ± 8 to 355 ± 14 g (*p* < 0.05). In the Tx group from Day 1 to Day 10, the body weight dropped from 338 ± 7 to 315 ± 11 (*p* *<* 0.05) g and subsequently increased to 329 ± 13 g at the end. In the thyroidectomy with T_4_ replacement (Tx + T4) group, the body weight dropped from 339 ± 13 to 296 ± 15 g (*p <* 0.05). In the PTU group, the rats had the highest steady body weight of 369 ± 6 g at the beginning and 380 ± 9 g at the end.

**Figure 2:**
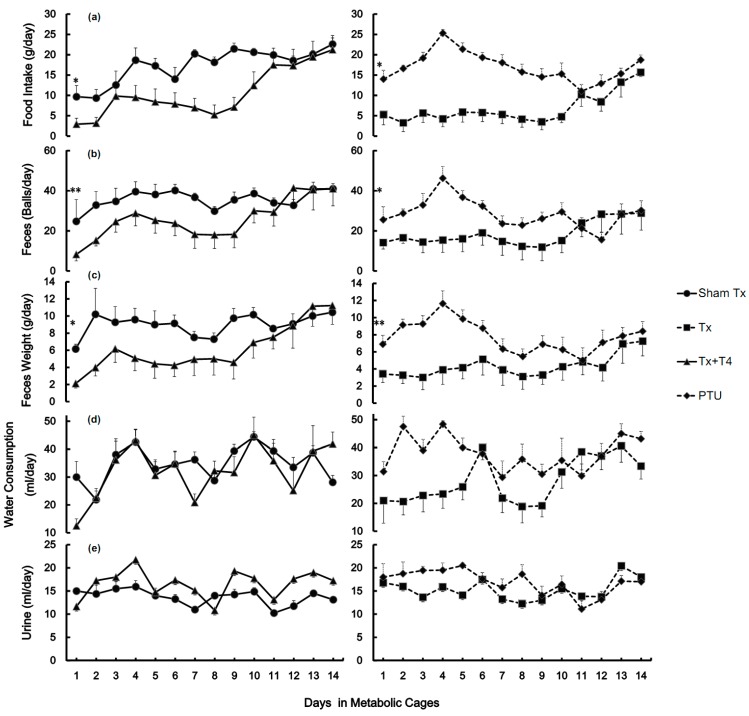
The metabolic profiles of rats measured after housing in metabolic cages (N = 8). The amount of food intake (**a**), feces balls (**b**), feces weight (**c**), water consumption (**d**), and urine output (**e**) were measured daily. Each figure is divided into 2 columns by means of hypothyroidism or not. The left column demonstrates non-hypothyroid groups such as Sham Tx and Tx + T4 groups whereas the right column demonstrates hypothyroid groups such as Tx and PTU groups. * *p* < 0.05, ** *p* < 0.01 compared with the other group on the same block of figure.

Water consumption and urine output varied daily with no consistent increases or decreases in any group (Figure 2d,e). The amount of food intake in the Sham Tx group increased from 9.7 ± 2.7 to 22.6 ± 1.5 g/d (*p <* 0.01) (Figure 2a). The number of feces balls increased non-significantly from 24.8 ± 10.9 to 41.0 ± 2.6 balls/d (Figure 2b), and feces weight increased from 6.2 ± 0.6 to 10.4 ± 0.5 g/d (*p <* 0.01) (Figure 2c). In the Tx group, the values of food intake slowly increased from 5.3 ± 2.5 to 15.7 ± 1.1 g/d (*p <* 0.01) (Figure 2b). Feces balls and weight increased from 14.2 ± 3.7 to 28.9 ± 5.7 balls/d (*p* < 0.01) (Figure 2b) and 3.4 ± 1.0 to 7.3 ± 1.7 g/d (Figure 2c), respectively. In the Tx + T4 group, the amount of food intake, feces balls, and feces weight increased substantially from 2.9 ± 1.5 to 21.2 ± 3.5 g/d (*p <* 0.01) (Figure 2a), 8.3 ± 3.2 to 41.0 ± 8.5 balls/d (*p <* 0.01) (Figure 2b), and 2.1± 0.5 to 11.2 ± 2.2 g/d (*p <* 0.01) (Figure 2c), respectively.

In the PTU group, the food intake, feces balls, and feces weight increased considerably to the highest values by Day 4, from 14.0 ± 2.2 to 25.3 ± 0.9 g/d (*p <* 0.01) (Figure 2a), 25.6 ± 6.5 to 46.3 ± 5.8 balls/d (*p <* 0.05) (Figure 2b), and 6.9 ± 1.0 to 11.6 ± 1.5 g/d (*p <* 0.05) (Figure 2c), respectively. After Day 4, all of the values dropped and approached those of the Tx group. At the end, the values were 18.7 ± 1.2 g/d (*p <* 0.05, compared with Day 1) (Figure 2a), 30.2 ± 4.8 balls/d (Figure 2b), and 8.4 ± 1.1 g/d (Figure 2c).

### 2.2. Effects of Hypothyroidism on Thyroid-Stimulating Hormone (TSH)

**Figure 3:**
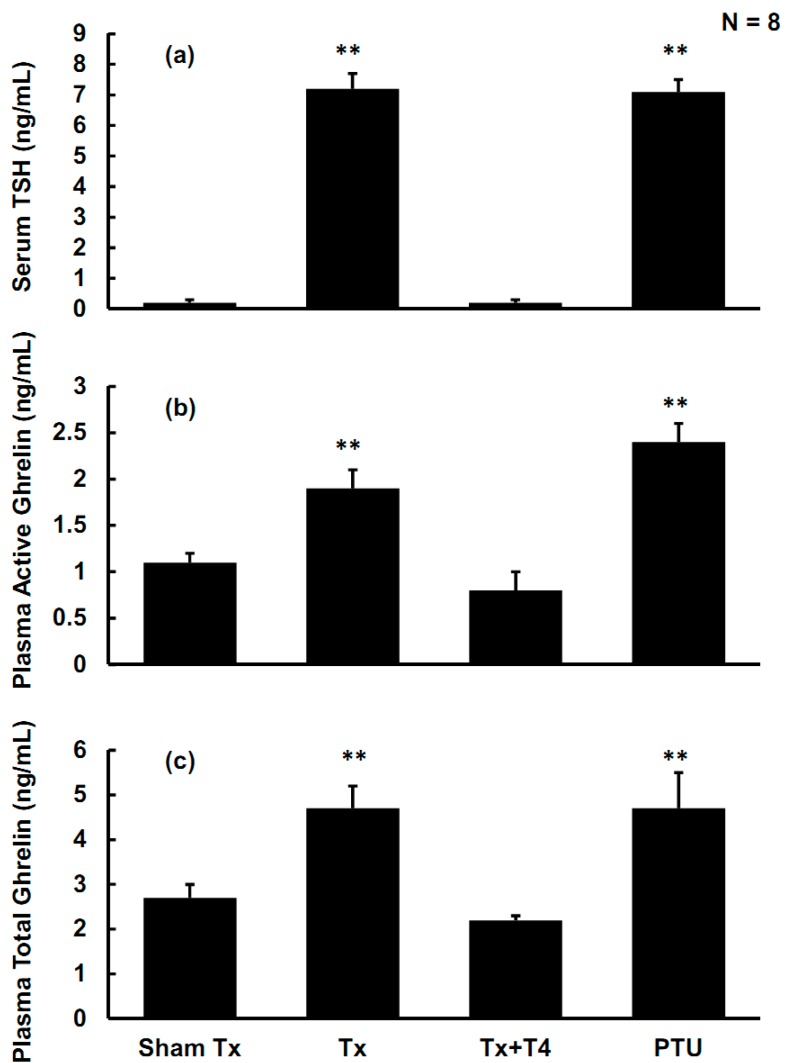
(**a**) The serum concentrations of the thyroid stimulating hormone (TSH) in rats. When the thyroid gland was destroyed by either thyroidectomy or PTU, the serum TSH levels elevated considerably, which indicated hypothyroidism in the Tx and PTU groups. (**b**) The plasma active ghrelin concentrations of rats. In the Tx and PTU groups, the plasma active ghrelin levels elevated significantly, whereas T_4_ replacement suppressed the levels of active ghrelin similar to that of the Sham Tx group. (**c**) The plasma total ghrelin concentrations of rats. In the Tx and PTU groups, the plasma total ghrelin levels are elevated. In the Tx + T4 group, administration of T_4_ suppressed the levels of total ghrelin to that of the Sham Tx group.

The average serum levels of the thyroid-stimulating hormone (TSH) in the Sham Tx group were 0.2 ± 0.1 ng/mL. However, in the Tx group and PTU group, the average serum TSH levels were as high as 7.2 ± 0.5 ng/mL and 7.1 ± 0.4 ng/mL, respectively (*p <* 0.01, compared with Sham Tx group). The results indicated that, in the Tx and PTU groups, the thyroid hormone was low and considerable TSH was secreted to compensate. In the Tx + T4 group, the TSH levels were suppressed by T_4_ replacement to 0.2 ± 0.1 ng/mL, which were close to those of the Sham Tx group (Figure 3a).

** *p* < 0.01 compared with the Sham Tx group.

### 2.3. Effects of Hypothyroidism on Active Ghrelin, Total Ghrelin, and Ghrelin Receptors in the Anterior Pituitary (AP)

**Figure 4:**
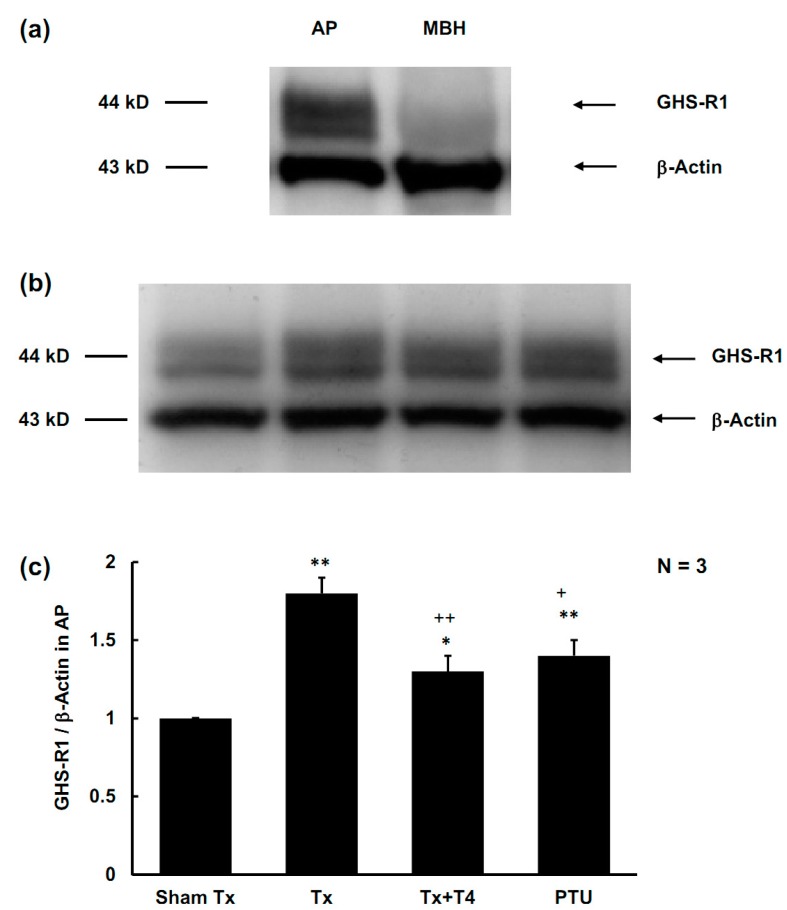
The GHS-R1 expression in the anterior pituitary gland (AP). (**a**) Specific expression of GHS-R1 presented as western blotting. The GHS-R1 expressed only in anterior pituitary (AP) but not medial basal hypothalamus (MBH). (**b**) Expression of GHS-R1 presented as western blotting in AP. (**c**) Expression of GHS-R1 in AP represented as GHS-R1/β-Actin ratio. In the Tx and PTU groups, the expression of GHS-R1 was enhanced. Replacement of T_4_ partially suppressed the GHS-R1 expression in AP.

The average plasma levels of active ghrelin in the Sham Tx group were 1.1 ± 0.1 ng/mL. In the Tx group and PTU group, the active ghrelin levels were as high as 1.9 ± 0.2 ng/mL and 2.4 ± 0.2 ng/mL, respectively (*p <* 0.01, compared with Sham Tx group). In the Tx + T4 group, the average plasma active ghrelin levels were suppressed by T_4_ replacement to 0.8 ± 0.2 ng/mL, which was non-significant compared to that of the Sham Tx group (Figure 4).

* *p* < 0.05, ** *p* < 0.01 compared with the Sham Tx group. + *p* < 0.05, + + *p* < 0.01 compared with the Tx group.

The average plasma levels of total ghrelin in the Sham Tx group were 2.7 ± 0.3 ng/mL. In the Tx and PTU groups, the total ghrelin levels were as high as 4.7 ± 0.5 ng/mL and 4.7 ± 0.8 ng/mL, respectively (*p <* 0.01, compared with the Sham Tx group). In the Tx + T4 group, the average plasma total ghrelin levels were suppressed again by T_4_ replacement to 2.2 ± 0.1 ng/mL, which was non-significant compared to the Sham Tx group (Figure 3b,c).

The GHS-R1 expressed in AP, but not in the medial basal hypothalamus (MBH) (Figure 4a). In the Tx group, the expression of GHS-R1 was 1.8 ± 0.1 times higher than that of the Sham Tx group (*p <* 0.01). In the PTU group, the expression of GHS-R1 was 1.4 ± 0.1 times higher than that of the Sham Tx group (*p <* 0.05). In the Tx + T4 group, the expression of GHS-R1 decreased to 1.3 ± 0.1; however, it differed from that of the Sham Tx group (*p <* 0.05) (Figure 4b,c).

### 2.4. Effects of Hypothyroidism on Growth Hormone (GH) and Insulin-Like Growth Factor-1 (IGF-1)

**Figure 5:**
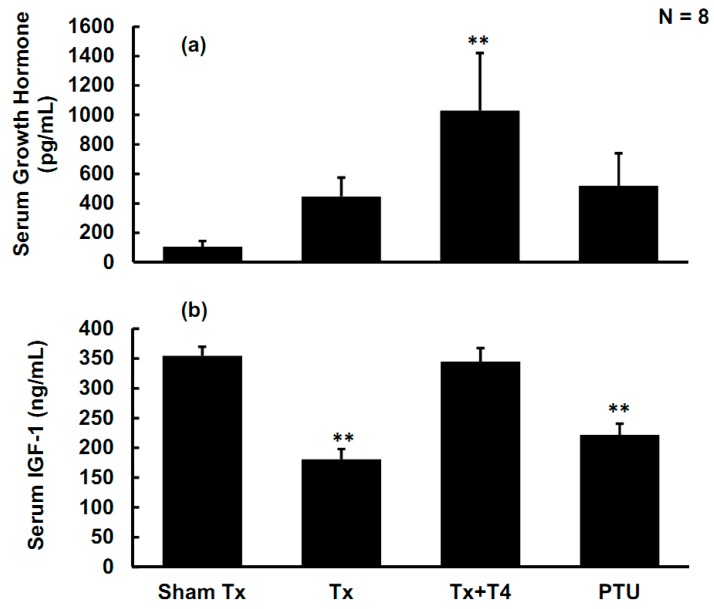
(**a**) The serum GH concentrations of rats. The GH concentration was elevated in the Tx, Tx + T4, and PTU groups; only the Tx + T4 group significantly differed from that of the Sham Tx group. (**b**) The IGF-1 concentrations in all rats. The IGF-1 concentration decreased remarkably in the Tx, and PTU groups and recovered to control levels by T_4_ administration.

The average serum levels of GH were 105.2 ± 39.0, 447.1 ± 127.7, 1029.7 ± 391.4, and 519.9 ± 219.6 pg/mL in the Sham Tx, Tx, Tx + T4, and PTU groups, respectively. Compared with the Sham Tx group, the average serum levels of GH were elevated in the Tx, Tx + T4, and PTU groups. However, the variation of GH levels were large in those groups, and only the levels of the Tx + T4 group differed from that of the Sham Tx group (*p <* 0.01) (Figure 5a).

The average serum IGF-1 levels in the Sham Tx group were as high as 354.6 ± 15.0 ng/mL. In the Tx and PTU groups, the average serum IGF-1 levels decreased considerably to 180.8 ± 17.3 and 221.8 ± 18.8 ng/mL, respectively (*p <* 0.01). Administering T_4_ to the Tx + T4 group successfully elevated the average IGF-1 levels to 344.7 ± 22.6 ng/mL, which differed from that of the Tx and PTU group (*p <* 0.01) and reached the levels of the Sham Tx group (Figure 5b).

** *p* < 0.01 compared with the Sham Tx group.

### 2.5. Relationship among TSH, Active Ghrelin, Total Ghrelin, GH, and IGF-1

**Figure 6:**
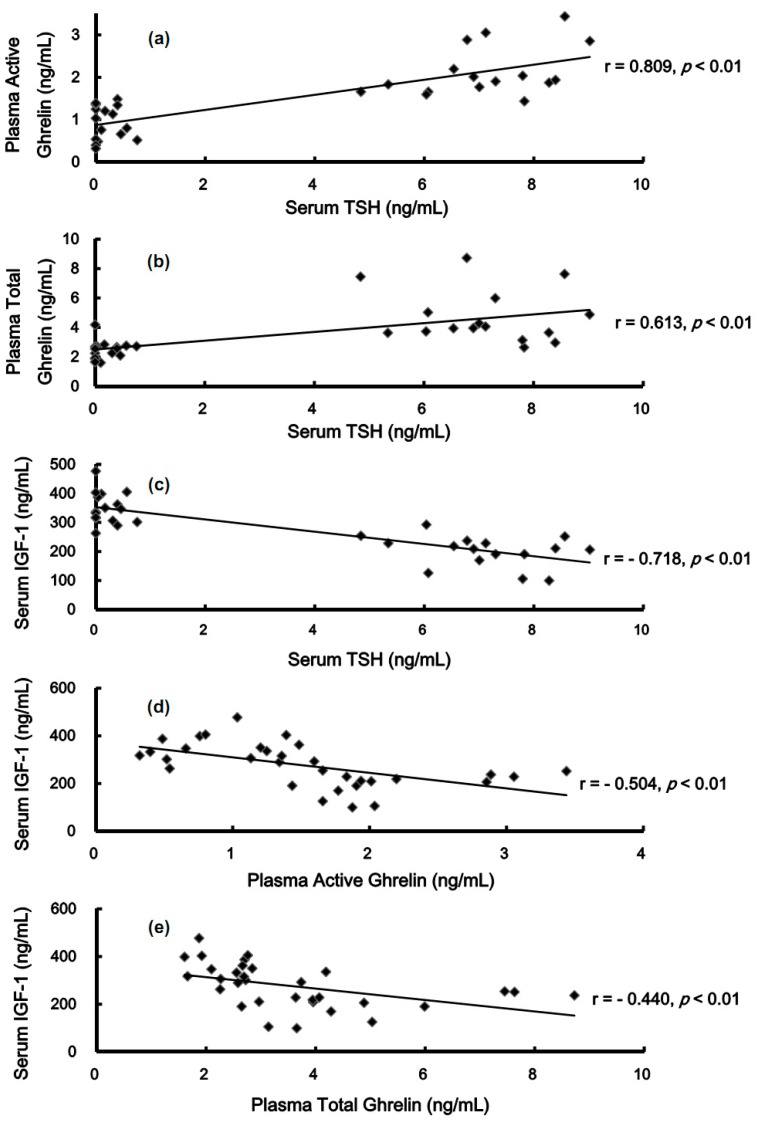
Relationship among TSH, active ghrelin, total ghrelin, GH, and IGH-1 in rats. N = 32 for the whole cohort. Serum TSH levels positively correlated with plasma active ghrelin and total ghrelin levels (**a**, **b**). Conversely, serum IGF-1 levels negatively correlated with plasma TSH, active ghrelin, and total ghrelin levels (**c**, **d**, **e**).

As shown in Figure 6, among the whole cohort, the TSH levels were positively related to active ghrelin and total ghrelin (*p <* 0.01) (Figure 6a,b) but negatively related to IGF-1 (*p <* 0.01) (Figure 6c). IGF-1 was negatively related to active ghrelin and total ghrelin (*p <* 0.01) (Figure 6d,e). There was no relationship between ghrelin and GH or between GH and IGF-1.

### 2.6. Relationship of Food Intake/Body Weight (BW) to Active Ghrelin, TSH, and IGF-1

**Figure 7:**
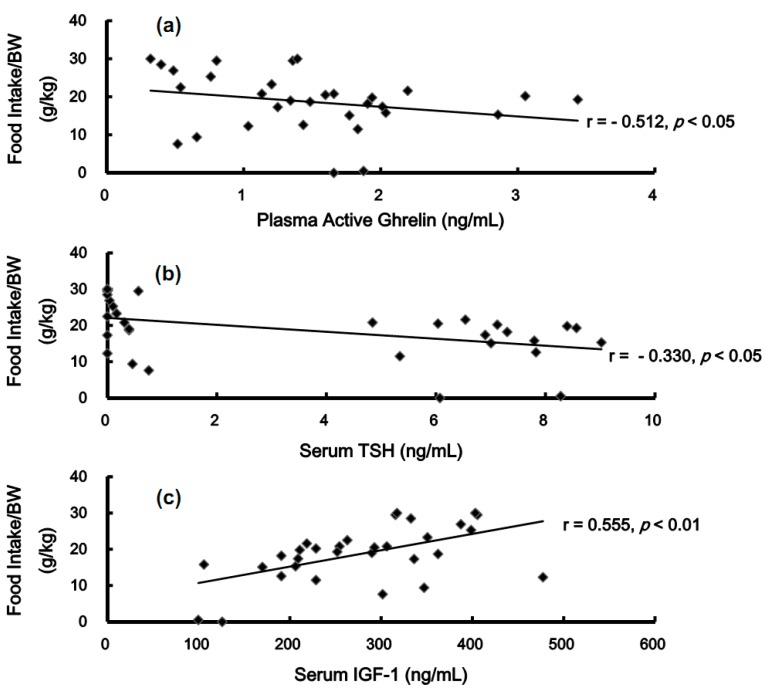
Relationship of food intake/body weight (BW) against active ghrelin, TSH, and IGF-1 in rats. N = 32 for the whole cohort. The food intake/BW negatively correlated with plasma active ghrelin and serum TSH levels (**a**, **b**). In contrast, it negatively correlated with IGF-1 serum levels (**c**).

Results of correlating the food intake/body weight to TSH, active ghrelin, total ghrelin, GH, and IGF-1 (Figure 7) revealed that the food intake/BW of all rats negatively correlated with plasma levels of active ghrelin (*p* < 0.05) (Figure 7a) and serum levels of TSH (*p* < 0.05) (Figure 7b). Conversely, the food intake/BW positively correlated with serum levels of IGF-1 (*p* < 0.01) (Figure 7c).

### 2.7. Discussion

After surgery, the rats in the Sham Tx, Tx, and Tx + T4 groups were observed and allowed to rest for 3 days before injection of a vehicle or T_4_. In the PTU group, the thyroid function was destroyed chemically and no surgery was performed. Therefore, the Tx group and PTU group can be easily compared to eradicate the effects of surgeries or chemicals. As shown in our results, the results showed that the hormone profiles of the Tx and PTU groups were consistent. According to the definition from the McGraw-Hill Concise Dictionary of Modern Medicine, the conditions in the experiment over the two weeks period can be referred to as subacute conditions. T3 or T4 levels were not measured because T3 and T4 have bound and unbound form with thyroxine-binding globulin. The thyroxine-binding globulin is easily interfered by corticosteroid levels [28]. TSH levels have been widely applied in clinical practice to represent the thyroid function directly [29], thus TSH levels were measured in the present study.

Following the 3 days resting period, the four groups of rats lived in metabolic cages for 14 days before being sacrificed. During this period, the Sham Tx group rats gained 10% weight (*p <* 0.05). The BW of the Tx group decreased by 8% during the first 10 days (*p* < 0.05) and subsequently increased. The BW of the Tx + T4 group decreased by 13% during the monitoring period (*p* < 0.05). The body weights of rats in the PTU group were maintained with minimal increase (Figure 1). The loss in body weight among the Sham Tx, Tx, and Tx + T4 groups within several days after surgery is reasonable. Body weights eventually increased in the Sham Tx and Tx groups, reflecting the recovery from the post-operatic stage. Despite the injected dose of T4 20 μg/kg correcting the hypothyroid condition, the body weight of the Tx + T4 group decreased continuously. This may be a result of an increase in the basal metabolic rate due to unregulated circulating of T_4_, thus reducing body weight while increasing food intake. The food intake of the Sham Tx, Tx, Tx + T4, and PTU groups increased 2.3 (*p* < 0.01), 3.0 (*p* < 0.01), 7.2 (*p* < 0.01), and 1.3 (*p* < 0.05)-fold, respectively, during monitoring period (Figure 2a). The Tx + T4 had the highest increase of food intake. Thyroid hormones not only increase the basal metabolic rate, but also subject appetite. Ishii *et al.* reported that subcutaneous injection of T_3_ 4.5 nmol/kg to male rats induced hyperphagia through phosphorylating hypothalamic AMPK, whereas the AMPK inhibitor compound C blocked the T_3_-induced hyperphagia [30].

In the beginning of the experiment, the amount of food intake among Sham Tx and PTU groups was similar, as well as that of the Tx and Tx + T4 groups. However, the line plot of the PTU group declined after Day 4, intersected with the Tx group on Day 11, and subsequently approached the Tx group. In contrary, the line plot of food intake in the Tx + T4 group intersected with that of the Sham Tx group on Day 11 and subsequently proceeded along with that of the Sham Tx group (Figure 2a). Similar trends were also found for the number of feces balls and feces weight (Figure 2b,c). Apparently, these results reveal that in the first days of observation, the food intake/feces behaviors were similar between minor-surgery (Sham Tx) and non-surgery (PTU) groups and between major-surgery (Tx and Tx + T4) groups. During the last 4 days before being sacrificed, the food intake/feces behaviors of true euthyroid (Sham Tx) and non-hypothyroid (Tx + T4) groups became similar, as well as those of hypothyroid (Tx and PTU) groups. It is reasonable that hypothyroid rats in the Tx and PTU groups had similar metabolic features, as well as those of non-hypothyroid rats in the subacute stage. Interestingly, in the PTU group, the amount of food intake and fecal excreta increased in the first 4 days, followed by a substantial decrease until Day 12. This phenomenon demonstrated that the PTU gradually destroyed thyroid gland and the residual thyroid hormones persisted in physiological functions for 8 days before vanishing.

Pu *et al.* found that PTU inhibits the production of aldosterone through the cAMP, Ca^2+^, and steroidogenic enzymes activities in male rats [11]. Hsu *et al.* also found that the release of atrial natriuretic peptide induced by 165 mM of sodium ion decreased in PTU-induced hypothyroid rats [31]. However, surgery or PTU injection had no obvious effects on water consumption or urine output in all groups of the present study (Figure 2d,e).

In 1999, Kojima *et al.* purified *n*-octanoyl ghrelin from rat stomachs and examined its effects on *in vivo* dispersed anterior pituitary cells in rats. The authors found that *n*-octanoyl ghrelin is the active form, and is the most potent ligand for growth-hormone secretagogue receptors (GHS-R) to stimulate the secretion of GH by increasing intracellular calcium concentration. The GH-releasing activity of ghrelin is as potent as that of the hypothalamic growth-hormone-releasing hormone (GHRH) [14]. The ghrelin/GHS-R axis [32,33] possesses diverse physiological functions, such as stimulating secretion of GH and enhancing food intake. Tschöp *et al.* reported that intracerebroventricular administration of ghrelin 1.2 nmol/kg/day and 12 nmol/kg/day for 7 days demonstrated ghrelin-dose-dependent hyperphagia in rats. Wren *et al.* infused ghrelin 5.0 pmol/kg/min intravenously to non-obese human subjects for 270 min. The infusion of ghrelin increased energy intake by 28% ± 3.9% [34]. In the hypothyroidism groups of Tx and PTU, the active ghrelin increased by 79% (*p* < 0.01) and 124% (*p* < 0.01), respectively, and the total ghrelin increased by 75% (*p* < 0.01) and 75% (*p* < 0.01), respectively. T_4_ replacement suppressed active ghrelin and total ghrelin levels to those of the Sham Tx group (Figure 3b,c). The serum TSH levels were positively correlated with plasma active ghrelin and total ghrelin (Figure 6a,b). Hypothyroidism also up-regulated the expression of GHS-R1 in the anterior pituitary, whereas T_4_ replacement partially suppressed the expression of GHS-R1 (Figure 4b,c). Total excision of thyroid gland in rats would cut off parathyroid gland and calcitonin-secreting cells at the same time, and circulating calcium homeostasis will be deteriorated. Apparently, GHS-R was substantially affected by T4 but the effects were partially interfered. The most possible cause for this interference is hypocalcemia that certainly occurs in total thyroidectomy rats. Kosowicz *et al.* compared 24 thyrotoxicosis patients, 25 hypothyroid patients after total thyroidectomy, and 17 normal subjects. The results revealed that hyperthyroid patients had higher circulating ghrelin levels, whereas hypothyroid patients had lower circulating ghrelin levels [35]. Several articles have presented similar findings [36,37], which are consistent with the results obtained in the present study.

As described above, the two major physiological functions of the ghrelin/GHS-R axis are stimulating secretion of GH and enhancing food intake. However, in the current study, the increased ghrelin levels and up-regulation of its receptors in hypothyroid rats did not substantially enhance the secretion of GH. By contrast, the Tx + T4 group achieved the highest levels of GH (*p* < 0.05) (Figure 5a). T3 was reported to stimulate GH release in rats [38], but to inhibit GH release in humans [39] and in bovines [40]. This suggests that action of the thyroid hormones on GH various among mammalian species. Apparently, the ghrelin/GHS-R axis is interfered with in the present study. Boulenger *et al.* reported that GHRH receptors in the anterior pituitary were down-regulated in hypothyroid rats [41]. Mulloy *et al.* found that intraperitoneal T_4_ (20 μg/kg/day) increased GH synthesis and GHRH-related GH release in Tx rats [42]. Therefore, alterations to functions of GHRH or its receptors in the anterior pituitary might surpass the ghrelin/GHS-R effects on GH secretion observed in the current study. Further, the food intake/BW negatively correlated with plasma active ghrelin (Figure 7a) and serum TSH levels (Figure 7b). Interestingly, the value of food intake/BW positively correlated with the serum levels of IGF-1 (Figure 7c). Further experiments should be performed to investigate this relationship. The hyperphagic effects of ghrelin seem to be overwhelmed by the hypothyroidism or increased IGF-1. Altogether, although hypothyroidism elevates the plasma ghrelin, this study presents novel finding that hypothyroidism disrupts the ghrelin/GHS-R axis in stimulating GH secretion and increasing food intake.

The GH/IGF-1 axis refers to the primary binding of circulating GH binds to its receptors in the liver, leading to the production of IGF-1, IGF-1 binding protein 3 (IGFBP-3), and other proteins through the JAK2-STAT pathway, the PI3K/Akt pathway, and the RAS/MAPK pathway [43]. Surprisingly, our results revealed that IGF-1 serum levels decreased by 51% (*p* < 0.01) and 63% (*p* < 0.01) in the Tx and PTU groups, respectively, and replacing T_4_ in the Tx + T4 group successfully increased the IGF-1 levels to control levels. The serum levels of IGF-1 did not correlate with its major stimulator, GH. On the contrary, the IGF-1 serum levels negatively correlated with serum or plasma levels of TSH (Figure 6c), active ghrelin (Figure 6d), and total ghrelin (Figure 6e). Romero *et al.* compared hypothyroid (thyroidectomized), thyroxine-treated thyroidectomized, and euthyroid control rats and found that the GH receptor mRNA in the liver decreased in male rats [44]. The changes could be prevented by thyroxine administration. Therefore, the decrease of GH receptors in the liver might cause the elimination of the release of IGF-1. Akin *et al.* investigated subclinical hypothyroid and subclinical hyperthyroid patients, among whom IGF-1 serum concentration was lower in subclinical hypothyroid patients, but not in hyperthyroid patients. Levothyroxine administration in proper dosage corrected this abnormalities [45]. Schmid *et al.* also indicated that T_4_ therapy in patients with primary or central hypothyroidism elevates the serum concentration of IGF-1 [46]. All these articles further support our findings and the novel conclusion that the GH/IGF-1 axis is disrupted again in hypothyroid rats.

## 3. Experimental Section

### 3.1. Animals

Male Sprague-Dawley rats aged 8 weeks weighing 350–400 g were housed in a temperature-controlled room (22 ± 1 °C) with 14 h of artificial illumination daily (06:00 AM to 8:00 PM). Food and water were provided *ad libitum*. The use of animals was approved by the Institutional Animal Care and Use Committee of National Yang-Ming University. All animals received human care in compliance with the principles of the Committee.

### 3.2. Chemicals, Antibodies, and Assay Kits

Triton X-100 was obtained from Riedel-deHaen (56029, Seelze, Germany) and sodium azide was obtained from Kokusan Chemical Works (Tokyo, Japan). L-Thyroxine (T2376), 6-propyl-2-thiouracil (PTU), bovine serum albumin (crystallized, A-4378, and RIA grade, A-7888), potassium chloride (P-4504), sodium chloride, IGEPAL^®^ CA-630, sodium deoxycholate, sodium dodecyl sulfate (SDS), tris hydrochloride (Tris), protease inhibitor cocktail, and boric acid (B-6768) were obtained from Sigma (St. Louis, MO, USA). The active ghrelin (GHRA-88HK) and total ghrelin (GHRA-89HK) kits were obtained from Linco Research (St. Charles, MS, USA). Growth hormone (GH) ELISA kits (KAP1081) and thyroid-stimulating hormone (TSH) IRMA kits (KIP1891) were purchased from Diasource (Nivelles, Belgium). The insulin-like growth factor-1 (IGF-1) ELISA Kit (EMI1001-1) was purchased from Assaypro (St. Charles, MO, USA). The antibodies against GHS-R1 (SC-10362) were purchased from Santa Cruz Biotechnology (Santa Cruz, CA, USA). The secondary rabbit antibodies against goat IgG (AP106P) were purchased from Millipore (Darmstadt, Germany). The protein molecular weight standard (SM0671) for western blotting was purchased from Fermentas International Incorporated (Burlinton, ON, Canada).

### 3.3. In Vivo Experiments: Hypothyroidism Induction and Thyroxine Replacement

The thyroidectomy and sham thyroidectomy procedures were performed as described previously [6]. In the sham thyroidectomy group (Sham Tx), the thyroid glands of ether-anesthetized rats were touched using cotton swabs. In the thyroidectomy group (Tx), the two lobes of the thyroid glands were carefully removed to preserve the capsule with the embedded parathyroid glands. The post-operated rats were observed for 3 days. Subsequently, all rats were housed in metabolic cages for 2 weeks. The rats of the Sham Tx and Tx groups were injected subcutaneously with 1 mL/kg alkaline saline with pH 11.0 at 9:00 AM daily. In the thyroxine replacement group (Tx + T4), we dissolved l-thyroxine in alkaline saline to yield a concentration of 20 μg/mL. The thyroidectomized rats were subcutaneously injected with 1 mL/kg of T_4_ saline at 9:00 AM daily to correct the hypothyroid status. In the 6-propyl-2-thiouracil treatment group (PTU), we dissolved PTU in alkaline saline to yield a concentration of 20 mg/mL. The rats that did not undergo an operation were subcutaneously injected with 1 mL/kg of PTU saline at 9:00 AM daily starting from the day when thyroidectomy was performed on the other groups.

The rats were decapitated at 9:00 AM after the final injections. Trunk blood samples were collected as serum for following TSH, growth hormone assays, and IGF-1 or plasma for active ghrelin and total ghrelin assays. The rat medial basal hypothalamus (MBH) block was dissected from the region between the rostral borders of the mammillary bodies and optic chiasma with a maximal depth of 2 mm. The anterior pituitary glands (AP) were also collected.

### 3.4. Western Blotting

The blocks of MBH and AP were homogenized in 200 μL of a lysis buffer (1.5% Na-lauroyl-sacrosine, 2.5 mM Tris base, 1 mM EDTA, and 0.1% PMSF, pH 7.8), and the protein was extracted. The aliquots (50 μg protein) of tissue lysates were used for electrophoresis on a 12% mini gel using standard SDS-PAGE procedures, and electrotransferred to polyvinylidene difluoride (PVDF) membranes (NEN Life Science Products, Boston, MA, USA) using a semi-dry transfer cell (Bio-Rad). The membranes were blocked in a blocking buffer (TBS-T buffer containing 5% skim milk) at room temperature for 120 min. The membranes were subsequently incubated with anti-GHS-R1 (1:1000) and anti-β-actin (1:10,000) overnight. After being washed 3 times with a TBS-T buffer for 5 min, the membranes were incubated with a horseradish peroxidase-conjugated secondary antibody (1:10000) for 2 h. Specific signals were detected using chemiluminescence (ECL, western blotting detection reagents, Amersham International, Buckinghamshire, UK). The bands located on the molecular weight of 44 kDa indicated GHS-R1, and those on 43 kDa indicated β-actin for internal control. The bands from western blotting were analyzed using ImageJ software (Version 1.45S, National Institutes of Health, USA). The integrated density in pixels of GHS-R1 was divided by that of β-actin. The quotient of GHS-R1/β-actin in the Sham Tx group was set to 1.

### 3.5. Statistical Analysis

All data are presented as mean ± SEM. SPSS 15.0 (IBM, Armonk, NY, USA) was used for statistical analyses [47]. The Student’s *t*-test was used for comparison between the two groups. The data from the four groups were processed using one-way analysis of variance (ANOVA) with *post-hoc* comparisons by Fisher's least significant difference (LSD). Pearson correlation coefficients were used to evaluate the relationship between hormones. Differences among groups were considered significant when *p* < 0.05 and highly significant when *p* < 0.01.

## 4. Conclusions

Surgical and chemical-induced hypothyroid rats had lower food intake and a steady body weight. In regard to growth-related molecules, the active ghrelin and total ghrelin secretion was enhanced and the expression of GHS-R was up-regulated in hypothyroid rats. However, IGF-1 secretion was inhibited by hypothyroidism. Replacement of T_4_ in the hypothyroid rats increased food intake and reduced body weight. The secretion of active ghrelin, total ghrelin, and GHS-R expression were suppressed by the administration of T_4_. The physiological functions of the ghrelin/GHS-R axis, namely stimulating GH release and increasing food intake, are interrupted, while the GH/IGF-1 axis operates independently from hypothyroidism.
